# Bacterial Communities Associated With Four Blooming Scyphozoan Jellyfish: Potential Species-Specific Consequences for Marine Organisms and Humans Health

**DOI:** 10.3389/fmicb.2021.647089

**Published:** 2021-05-05

**Authors:** Saijun Peng, Wenjin Hao, Yongxue Li, Lei Wang, Tingting Sun, Jianmin Zhao, Zhijun Dong

**Affiliations:** ^1^Muping Coastal Environment Research Station, Yantai Institute of Coastal Zone Research, Chinese Academy of Sciences, Yantai, China; ^2^University of Chinese Academy of Sciences, Beijing, China; ^3^School of Life Sciences, Nantong University, Nantong, China; ^4^Key Laboratory of Coastal Environmental Processes and Ecological Remediation, Yantai Institute of Coastal Zone Research, Chinese Academy of Sciences, Yantai, China; ^5^Center for Ocean Mega-Science, Chinese Academy of Sciences, Qingdao, China

**Keywords:** jellyfish blooms, scyphomedusae, jellyfish microbiome, pathogenic bacteria, high-throughput sequencing

## Abstract

Cnidarians have large surface areas available for colonization by microbial organisms, which serve a multitude of functions in the environment. However, relatively few studies have been conducted on scyphozoan-associated microbial communities. Blooms of scyphozoan species are common worldwide and can have numerous deleterious consequences on the marine ecosystem. Four scyphozoan species, *Aurelia coerulea*, *Cyanea nozakii*, *Nemopilema nomurai*, and *Rhopilema esculentum*, form large blooms in Chinese seas. In this study, we analyzed the bacterial communities associated with these four jellyfish based on 16S rRNA gene sequencing. We found that the bacterial communities associated with each scyphozoan species were significantly different from each other and from those of the surrounding seawater. There were no significant differences between the bacterial communities associated with different body parts of the four scyphozoan jellyfish. Core bacteria in various compartments of the four scyphozoan taxa comprised 57 OTUs (Operational Taxonomic Units), dominated by genera *Mycoplasma*, *Vibrio*, *Ralstonia*, *Tenacibaculum*, *Shingomonas* and *Phyllobacterium*. FAPROTAX function prediction revealed that jellyfish could influence microbially mediated biogeochemical cycles, compound degradation and transmit pathogens in regions where they proliferate. Finally, Six genera of potentially pathogenic bacteria associated with the scyphozoans were detected: *Vibrio*, *Mycoplasma*, *Ralstonia*, *Tenacibaculum*, *Nautella*, and *Acinetobacter*. Our study suggests that blooms of these four common scyphozoans may cause jellyfish species-specific impacts on element cycling in marine ecosystems, and serve as vectors of pathogenic bacteria to threaten other marine organisms and human health.

## Introduction

The external and internal surfaces of marine animals are considered to be ubiquitously colonized by microorganisms (Grossart and Tang, 2010). Microbial associations are often dominated by bacteria but co-inhabited by fungi, protozoa, archaea, and viruses (Cárdenas et al., 2020; Deines et al., 2020). Microbial colonization of a certain surface is determined by the availability of nutrients, host immune responses, and competition between microbes from the surrounding environment for attachment space (Wilson et al., 2011). Microbial communities have therefore developed special mechanisms to interfere with the ability of adverse bacteria to colonize surfaces and acquire nutrients (Vezzulli et al., 2012). Microorganisms are vital to marine ecology, being involved in a multitude of exchange processes with the environment: respiration, exudation of wastes and secondary metabolites, absorption of energetic irradiation or informational signals, uptake of nutrients, and gases, and so on (Wahl et al., 2010). Microbes may influence developmental programs of hosts with complex life history strategies, such as inducing normal developmental changes in morphology and maturation of the immune system (Schuh et al., 2020). Changes in composition and function of microbiomes have been linked to disorders in many organisms (McKenney and Pamer, 2015; Contijoch et al., 2019). A recent study suggested microbes could directly affect the functional activity of the nervous system by controlling spontaneous body contractions (Klimovich et al., 2020). Development of multicellularity in eukaryotes may never have been autonomous, rather requiring transient or persistent interactions with the microbial world (Bosch and McFall-Ngai, 2021).

Bacterial communities associated with jellyfish mainly consisted of Alpha- and Gammaproteobacteria, Bacteroidetes, Tenericutes, and Cyanobacteria (Weiland-Bräuer et al., 2015; Daley et al., 2016; Basso et al., 2019; Kramar et al., 2019; Jaspers et al., 2020; Stabili et al., 2020). Certain jellyfish increase in numbers and form blooms or aggregations during certain periods, during which the population density of the pelagic medusae peaks distinctly over broad temporal and regional scales (Purcell et al., 2007; Condon et al., 2012; Purcell, 2012). It is well documented that jellyfish as voracious predators may graze upon a variety of plankton taxa (Colin et al., 2010; Granhag et al., 2011) and cause cascading effects on the food web (Zoccarato et al., 2016; Xiao et al., 2019). Jellyfish can stimulate bacterioplankton growth by direct release of nutrients from tissue, mucus secretion, excretion, and release of food particles when feeding (Titelman et al., 2006; Pitt et al., 2009; Condon et al., 2012). Increased bacterial abundance has been observed in the vicinity of decaying jellyfish (Titelman et al., 2006; Tinta et al., 2010; Blanchet et al., 2015; Guy-Haim et al., 2020; Tinta et al., 2020), such as the genera *Vibrio* and *Pseudoalteromonas* (Tinta et al., 2012). However, even more importantly, growth of bacterioplankton surrounding live jellyfish may be stimulated by the release of nutrients and bioavailable carbon (Titelman et al., 2006; Tinta et al., 2010; Condon et al., 2011). Hence, bacterial communities associated with jellyfish need to be resolved in order to understand the interaction between jellyfish and marine microbial communities, and its impact on biogeochemical cycles and marine ecosystems.

Jellyfish-associated bacterial communities have been found to play an important role in the life cycle of jellyfish. Planulae and propagules of some scyphozoans have been successfully induced to enter metamorphosis using bacteria found in the environment of settled polyps; in particular, larvae of *Cassiopea andromeda* responded to a species of *Vibrio alginolyticus* (Neumann, 1979; Hofmann et al., 1996), and the propagules (pedal stolons) of *Aurelia aurita* to a species of Micrococcaceae (Schmahl, 1985). The asexual reproduction and strobilation of *A. aurita* was severely inhibited in the absence of the native microbiota (Weiland-Bräuer et al., 2020). Moreover, Lee et al. (2018) identified OTUs within *Chrysaora plocamia* and *A. aurita* most closely related to a suite of metabolically and physiologically diverse microorganisms capable of mediating the interrelated pathways of carbon, nitrogen, sulfur, and phosphorus. Several jellyfish-associated bacteria were previously also associated with the processing of peculiar substances, such as polycyclic aromatic hydrocarbons (PAHs), plastics, and xenobiotics found in the ocean, with possible benefits for the host. For example, Almeda et al. (2013) suggested the presence of PAH-degrading bacteria in *A. aurita*; Kramar et al. (2019) detected PAH and plastic-degrading bacteria (*Burkholderia*, *Achromobacter* and *Kocuria*) within the gastric cavity of *A. aurita*.

Recent studies have indicated that scyphozoan jellyfish may be key vectors of bacterial pathogens, and may thus harm cultured organisms and human well-being (Ferguson et al., 2010; Schuett and Doepke, 2010; Delannoy et al., 2011; Småge et al., 2017; Basso et al., 2019; Clinton et al., 2020). An outbreak of disease in farmed Atlantic salmon has been linked to *Tenacibaculum maritimum* isolated within the adherent microbial communities of the jellyfish *Phialella quadrata* and *Pelagia noctiluca* (Ferguson et al., 2010; Delannoy et al., 2011). Therefore, it is essential to identify the bacteria associated with the scyphozoans to understand the role scyphozoan jellyfish may play in the spread of bacterial disease. Recently, a number of potential pathogens of commercial aquaculture have been identified, including *Moritella viscosa* in the jellyfish *Cyanea lamarckii* (Schuett and Doepke, 2010); *Chryseobacterium*, *Flavobacterium, Tenacibaculum*, *Coxiella* and *Vibrio* in the blooming scyphozoan *Rhizostoma pulmo* (Basso et al., 2019); and *Aeromonas salmonicida*, *A. molluscorum*, *Pseudomonas fluorescens*, *P. fulva* and *Vibrio splendidus* in the Lion’s Mane jellyfish *Cyanea capillata* (Clinton et al., 2020).

The association of bacteria with jellyfish is highly dynamic and complex, specifically expressed as distinction between jellyfish microbiomes and the ambient water, as well as the population specificity, taxa specificity, life stages specificity and body part specificity of the microbial groups associated with jellyfish (Schuett and Doepke, 2010; Tinta et al., 2012; Cortés-Lara et al., 2015; Weiland-Bräuer et al., 2015; Viver et al., 2017; Lee et al., 2018; Basso et al., 2019; Hao et al., 2019; Kramar et al., 2019; Tinta et al., 2019). To the authors’ knowledge, no studies to date have focused on the microbiomes associated with jellyfish species in Chinese Seas. The present study focuses on the bacterial communities associated with *Aurelia coerulea*, *Cyanea nozakii* and *Nemopilema nomurai*, as the scyphozoans with the highest frequencies of outbreaks in Chinese seas (Dong et al., 2010; Wang P.P. et al., 2020), as well as *Rhopilema esculentum*, which is an economically valuable edible jellyfish and is artificially proliferated and released to increase its population. For example, the biomass of *A. coerulea* can reach 45.45 tons km^–2^ in Chinese local outbreak areas (Wang P.P. et al., 2020), *C. nozakii* has been proven to be able to reach a biomass of 4000-6000 individuals km^–2^ during bloom periods (Zhang et al., 2012), and *N. nomurai* during the outbreak periods may reach a maximum abundance of 15-75 tons km^–2^ in some China waters (Wang P.P. et al., 2020). The total harvest of *R. esculentum* peaked at 0.43 million tons in 1998 (Dong et al., 2014). The aims of our research were to characterize the differences and similarities of the bacterial communities associated with the four blooming scyphozoans in Chinese Seas based on 16S rRNA gene sequencing. Ecological functions and potentially pathogenic bacteria within scyphomedusa-associated bacterial communities are also discussed to provide insights into the potential consequences for marine organisms and humans health.

## Materials and Methods

### Sample Collection and Processing

**FIGURE 1:**
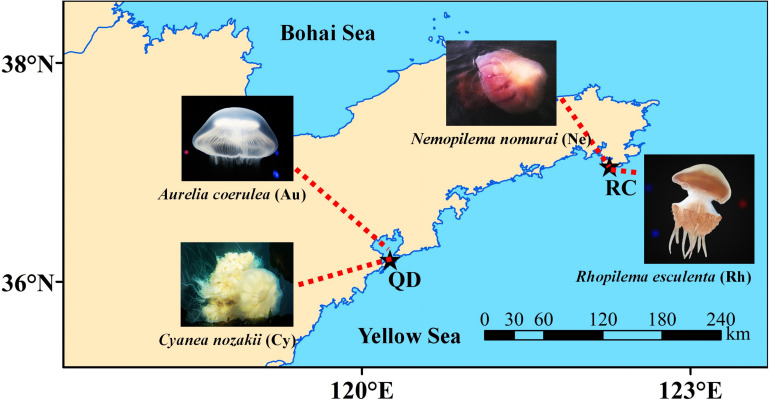
Sampling sites for four blooming scyphozoan jellyfish. **Note:** Abbreviations: QD, Jiaozhou Bay in Qingdao; RC, Shidao Bay in Rongcheng.

Scyphomedusa specimens were carefully collected from Jiaozhou Bay, Qingdao (120.298 E, 36.066 N; QD) and Shidao Bay, Rongcheng (122.414 E, 36.918 N; RC), in Shandong province, in the Northern Yellow Sea of China (Figure 1). All samples were collected at the peak abundance at which *A. coerulea* (Au) and *C. nozakii* (Cy) occurred in Qingdao, and *N. nomurai* (Ne) and *R. esculentum* (Rh) occurred in Rongcheng in August 2018 (Figure 1). Scyphomedusae were temporarily placed in 20 L aerated tanks with ambient seawater which were transported to the laboratory in 4 hours. Five samples of each species which had empty guts were individually washed three times with sterile seawater (0.22 μm pre-filtered and autoclaved) to remove loosely associated microorganisms and bacteria from the surrounding seawater. After rinsing, each individual was dissected into the following parts with sterilized scalpels: umbrella (U), oral arms (O), stomach (S) and gonad (G). Umbrella, oral arms and stomach were collected from the individuals of *A. coerulea*; umbrella, oral arms and gonad were collected from the other three scyphomedusa species (*C. nozakii*, *N. nomurai* and *R. esculentum*). Specimens were then given a final rinse with sterile seawater, flash-frozen with liquid nitrogen, and stored at −80°C. Bulk seawater samples (500 mL) collected at the same time as scyphomedusa collection from each location were subjected to sequential filtration. Particle-associated bacteria were removed with 3 μm-pore size filters (TCTP, 47 mm, Millipore, Germany). To collect free-living bacteria, the resulting filtrate was subsequently filtered on a 0.22 μm-pore size filter (GTTP, 47 mm, Millipore, Germany). This was repeated three times for each location to obtain bacteria from the water column for comparison with scyphomedusa-associated bacteria. In total, 60 scyphomedusa samples (4 species × 3 body parts × 5 individuals) and 6 seawater samples (2 locations × 3 replicates) were prepared for DNA extraction.

### DNA Extraction and 16S rRNA Gene Amplicon Sequencing

one hundred milli gram of each scyphomedusa sample were homogenized for bacterial DNA extraction using CTAB (cetyl-trimethyl-ammonium bromide), as described by Hao (2014) with slight modifications. The whole filter membranes of each seawater sample were used to extract the total DNA of seawater samples using a PowerWater DNA Isolation Kit (MOBIO, United States) according to the manufacturer’s instructions. DNA was diluted to 1 ng/μL using sterile water. The variable 4 and 5 regions of 16S rRNA genes were amplified used specific primers 515F (5′-GTG CCA GCM GCC GCG GTA A-3′) and 907R (5′-CCG TCA ATT CCT TTG AGT TT-3′) (Walters et al., 2015) with the forward primer modified to contain a unique 6 nt barcode at the 5′ end. All PCR reactions were carried out using the Phusion High-Fidelity PCR Master Mix (New England Biolabs) with 0.2 μM of each primer and 10 ng template DNA. Thermal cycling consisted of initial denaturation at 98°C for 1 min, followed by 30 cycles of denaturation at 98°C for 10 s, annealing at 50°C for 30 s, and elongation at 72°C for 30 s, and finally 72°C for 5 min. All PCRs were performed in triplicate, and no-template controls were included in all steps of the process. PCR products were detected by electrophoresis in a 2% (w/v) agarose gel. PCR amplicons of each sample with bright bands were mixed in equal density ratios and purified with GeneJET^TM^ Gel Extraction Kit (Thermo Scientific). The amplicon libraries were generated using Ion Plus Fragment Library Kit 48 rxns (Thermo Scientific) and sequenced using the Ion S5^TM^ XL platform at Novogene Bioinformatics Technology Co., Ltd. (Beijing, China).

### Sequence Analysis of the 16S rRNA Amplicons

Single-end reads were assigned to samples based on unique barcode and truncated by cutting off the barcode and primer sequence. Quality filtering on the raw reads was performed under specific filtering conditions to obtain the high-quality clean reads according to the Cutadapt (v1.9.1) quality control process (Martin, 2011). All raw reads were compared with the SILVA 132^1^ (Quast et al., 2013) using the UCHIME algorithm (Robert et al., 2011) to detect chimera sequences, and then the chimera sequences were removed to obtain the Clean Reads (Brian et al., 2011).

Sequences with ≥ 97% similarity were assigned to the same operational taxonomic units (OTUs) and the representative sequence for each OTU was screened for further annotation with Uparse software (v7.0.1001) (Robert, 2013). For each representative sequence, assignments of taxonomic annotations from the phylum to species levels to the clean OTU sequences were performed using the Silva Database based on the Mothur algorithm (Quast et al., 2013). The abundance data for the OTUs were normalized using a standard of sequence number corresponding to the sample with the least sequences. Then the original OTU data of the repeated samples of each body part of each scyphozoan as well as seawater samples were combined by averaging. Subsequently, the combined OTU data of the three body parts of each scyphozoan were averaged and merged to obtain the OTU data of a single total scyphozoan.

### Data Analysis

Multiple Alpha diversity indices (Observed OTUs, Chao1, ACE, Shannon, Simpson and Good’s coverage) were calculated based on 37847 reads per sample (minimum number of sequences required to normalize the differences in sequencing depth) using QIIME and a box plot of the Shannon index was visualized using R software (V3.6.2). Statistically significant differences of the Shannon indices among the microbiota of the four scyphozoan taxa, the microbiota of the scyphozoans and ambient seawater, and the microbiota of the three compartments of each scyphozoan taxa were set at *p* < 0.05 using a Wilcoxon test based on IBM SPSS Statistics software (V22). The Principal Co-ordinates Analysis (PCoA) was performed to determine the Beta diversity of samples based on unweighted Unifrac distance at OTU level and visualized using ggplot2 and vegan package of R software (V3.6.2). Permutational analysis of molecular variance (PERMANOVA) was conducted to test statistical differences in the Beta diversity of the microbiota of the four scyphozoan taxa, the microbiota of the scyphozoans and ambient seawater, and the microbiota of the three compartments of each scyphozoan taxa using Primer software (Version 6, PRIMER-E Ltd., Lutton, United Kingdom), with 999 random permutations of the appropriate units. The relative abundances of dominant bacteria at distinct classification levels among scyphozoan groups and seawater groups were demonstrated using stacked bar plots, and visualized with Origin software (Version Pro 2018). The core bacterial communities associated with all the studied body parts of the four scyphozoan taxa were selected based on the relative abundance information of the OTUs, shown in a bubble plot using R software (V3.6.2). Functional Annotation of Prokaryotic Taxa (FAPROTAX) (Louca et al., 2016) was applied to predict the main ecological functions of the microbial communities associated with four scyphozoan species and ambient seawater. The cumulative absolute abundances of the OTUs contributing to functional groups were square root transformed after normalization and visualized in a heat-map with R software (Version 3.6.2). Then a Mann-Whitney *U*-test was carried out to determine significantly different functional groups of bacteria between scyphozoan and seawater, while a Kruskal-Wallis test was conducted to verify significant differences between functional groups of the four scyphozoan species, both with IBM SPSS Statistics software (V22). A phylogenetic tree was constructed by the Neighbor-joining method using Mega software (Version X) based on the V4-V5 region of the 16S rRNA gene (Kumar et al., 2018). Sequence data from identified pathogenic strains deposited in the NCBI database for 16S rRNA gene were used in this study.

## Results

The bacterial communities associated with four scyphomedusae and surrounding seawater were analyzed by Ion S5^TM^ XL sequencing which generated 4,351,887 filtered high-quality sequences with an average of 69,078 sequences for each sample. In total, 1888 OTUs were defined by 97% sequence similarity, clustered into 2 Kingdoms (Bacteria and Archaea), 41 phyla, 53 classes, 115 orders, 228 families, and 547 genera. The coverage of all samples in this study was ≥ 99.7% and Shannon curves showed saturation for all samples, indicating good coverage of the bacterial communities in the present study (Supplementary Table 1 and Supplementary Figure 1). In the following analysis, only bacteria with a relative abundance higher than 1% in at least one group were considered.

### Bacterial Community Composition

**FIGURE 2:**
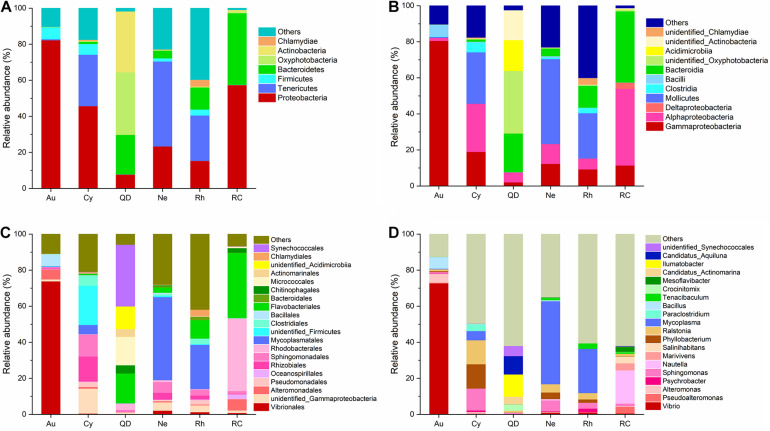
Stacked Bar plots of bacterial taxa at **(A)** phylum level, **(B)** class level, **(C)** order level, and **(D)** genus level, in scyphozoan-associated bacterial communities and free-living bacteria in surrounding seawater. **Note:** Abbreviations: Au, *A. coerulea* from Jiaozhou Bay in Qingdao; Cy, *C. nozakii* from Jiaozhou Bay in Qingdao; Ne, *N. nomurai* from Shidao Bay in Rongcheng; Rh, *R. esculentum* from Shidao Bay in Rongcheng; QD, ambient seawater from Jiaozhou Bay in Qingdao; RC, ambient seawater from Shidao Bay in Rongcheng. **(A)** Phylum and **(B)** class levels list taxa with mean relative abundance > 1% in at least one group, and those with mean relative abundance of < 1% across all groups were subsumed under “others.” **(C)** Order and **(D)** genus levels list the most abundant 20 bacterial taxa with mean relative abundance > 1% in at least one group, with less abundant taxa included under “others.”

The relative abundance of taxa identified to the most resolvable taxa (phylum, class, order, and genus) were shown as stacked bar plots for the analyzed groups (Figure 2). At the level of phylum and class, the bacterial communities associated with *A. coerulea* was dominated by Proteobacteria (82.27%), which mainly consisted of the class Gammaproteobacteria (80.57%), followed by Firmicutes (of which Bacilli was the most common class, 6.63%). Proteobacteria (45.75%) and Tenericutes (28.50%) were predominant in the bacterial communities of *C. nozakii*, with Gammaproteobacteria (19.04%) and Alphaproteobacteria (26.67%) the most common classes. Oxyphotobacteria (34.67%), Actinobacteria (33.79%), Bacteroidetes (21.94%) and Proteobacteria (7.78%) were most common in the surrounding seawater of Qingdao. Tenericutes were the most abundant bacteria in *N. nomurai* and *R. esculentum* with 47.10% and 25.14%, respectively, followed by Proteobacteria, accounting for 22.35% and 30.37%, respectively. Proteobacteria (57.46%) and Bacteroidetes (39.78%) were dominant in the surrounding seawater of Rongcheng.

At the level of order and genus, the most abundant bacteria in *A. coerulea* were Vibrionales (73.90%, dominated by genus *Vibrio*), Bacillales (6.48%, dominated by genus *Bacillus*) and Alteromonadales (5.31%, dominated by genus *Alteromonas*). Prevailing bacteria in *C. nozakii* were Rhizobiales (13.92%, dominated by genus *Phyllobacterium*), Sphingomonadales (12.16%, dominated by genus *Sphingomonas*), Clostridiales (5.46%, dominated by genus *Paraclostridium*), Mycoplasmatales (5.08%, dominated by genus *Mycoplasma*) and genus *Ralstonia* (13.28%). The ambient water at the Qingdao location contained mainly Synechococcales (34.22%), Flavobacteriales (16.53%), Micrococcales (15.79%), Chitinophagales (4.51%), Actinomarinales (4.22%) and Rhodobacterales (3.56%). The bacterial communities associated with *N. nomurai* and *R. esculentum* were relatively similar, with high relative abundances of order Mycoplasmatales, 46.04% and 24.54% respectively, of which *Mycoplasma* was the most abundant genus in both scyphozoans. Rhizobiales, Sphingomonadales (mostly genus *Sphingomonas*), Flavobacteriales (mostly genus *Tenacibaculum*), Vibrionales (mostly genus *Vibrio*), and genera *Phyllobacterium* and *Ralstonia* were also detected in *N. nomurai* and *R. esculentum*. In addition, the bacterial communities of the Rongcheng seawater were characterized by high relative abundances of order Rhodobacterales (40.33%), Flavobacteriales (36.17%), Alteromonadales (6.25%), Oceanospirillales (2.66%) and Chitinophagales (2.56%). The composition and relative abundance of bacterial communities in scyphomedusae were significantly different from those in the ambient seawater (Kruskal-Wallis test, *p* < 0.05) (Figure 2). Furthermore, the composition and relative abundance of the four scyphomedusa-associated bacterial communities were significantly different from each other (Kruskal-Wallis test, *p* < 0.05) (Figure 2).

### Bacterial Community Diversity

**FIGURE 3:**
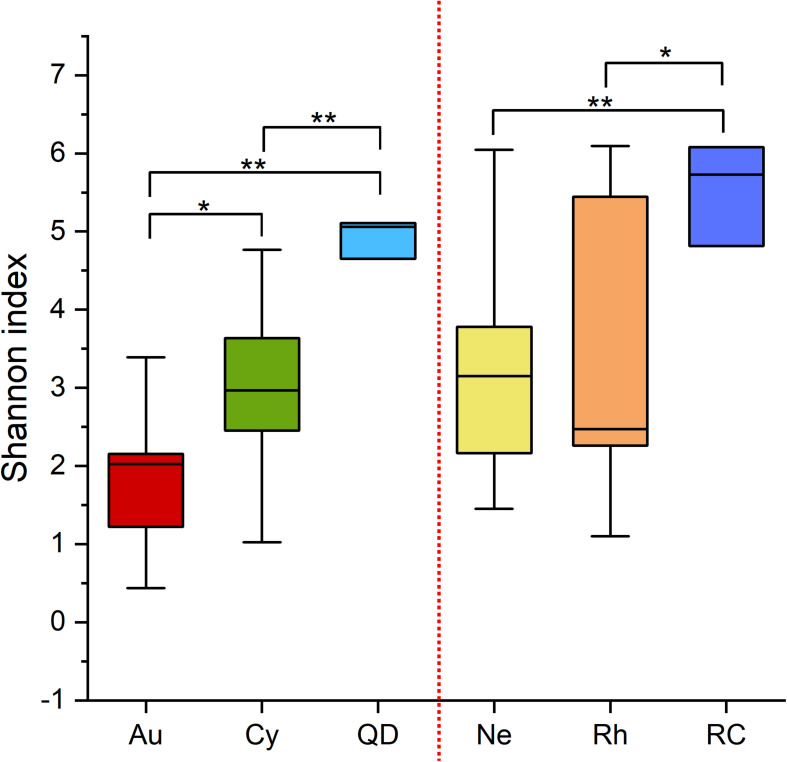
Boxplot of Shannon indices of scyphozoan-associated bacterial communities and free-living bacteria in surrounding seawater. **Note:** Abbreviations: Au, *A. coerulea* from Jiaozhou Bay in Qingdao; Cy, *C. nozakii* from Jiaozhou Bay in Qingdao; Ne, *N. nomurai* from Shidao Bay in Rongcheng; Rh, *R. esculentum* from Shidao Bay in Rongcheng; QD, ambient seawater from Jiaozhou Bay in Qingdao; RC, ambient seawater from Shidao Bay in Rongcheng. Significant differences were determined using the Wilcoxon test, *: *p* < 0.05, **: *p* < 0.01.

The α-diversities (Shannon indices) of the bacterial communities associated with the four scyphomedusae, *A. coerulea* (1.5), *C. nozakii* (2.4), *N. nomurai* (2.4) and *R. esculentum* (3.0), were lower than those of the water column sampled at both locations (5.3 in Qingdao and 5.8 in Rongcheng) according to the 16S rRNA gene sequencing. The statistical analysis showed significant differences between scyphomedusae and ambient seawater, and between scyphomedusae *A. coerulea* and *C. nozakii* (Wilcoxon test, *p* < 0.05) (Figure 3). However, there were no significant differences between the Shannon indices of scyphomedusae *N. nomurai* and *R. esculentum* in Rongcheng (Wilcoxon test, *p* > 0.05) (Figure 3).

**FIGURE 4:**
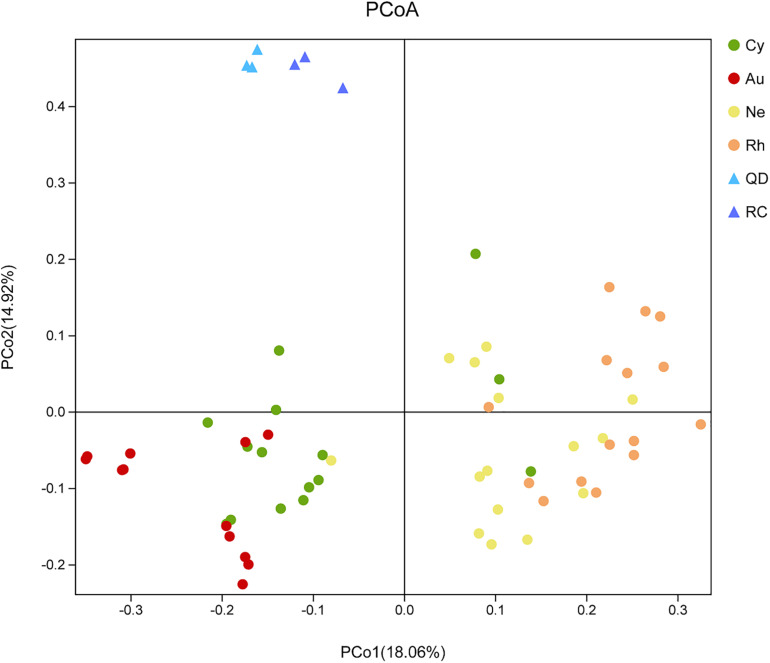
Unweighted unifrac PCoA plot of scyphozoan-associated bacterial communities and free-living bacteria in surrounding seawater. **Note:** Abbreviations: Au, *A. coerulea* from Jiaozhou Bay in Qingdao; Cy, *C. nozakii* from Jiaozhou Bay in Qingdao; Ne, *N. nomurai* from Shidao Bay in Rongcheng; Rh, *R. esculentum* from Shidao Bay in Rongcheng; QD, ambient seawater from Jiaozhou Bay in Qingdao; RC, ambient seawater from Shidao Bay in Rongcheng.

Principal Co-ordinates Analysis demonstrated that the bacterial communities associated with the four scyphomedusa taxa were significantly different from the free-living bacterial communities in the seawater at the two locations (Qingdao and Rongcheng) according to PERMANOVA main test (*p* < 0.01) (Figure 4 and Tables 1, 2). *A. coerulea* and *C. nozakii*-associated bacterial communities were relatively separate from those of *N. nomurai* and *R. esculentum*, while those of the two scyphomedusa species sampled from the same seawater were closely clustered. The significance analysis revealed that the bacterial communities associated with the four scyphomedusa taxa all had extremely significant differences from each other (PERMANOVA, *p* < 0.01) (Figure 4 and Tables 1, 2).

**TABLE 1:** The result of PERMANOVA analysis in all groups.

Group	Au vs. Cy vs. Ne vs. Rh vs. QD vs. RC
Sample size	60
pseudo-*F*	4.94
*P*-value	0.001

***Note:** Abbreviation: Au, A. coerulea from Jiaozhou Bay in Qingdao; Cy, C. nozakii from Jiaozhou Bay in Qingdao; Ne, N. nomurai from Shidao Bay in Rongcheng; Rh, R. esculentum from Shidao Bay in Rongcheng; QD, ambient seawater from Jiaozhou Bay in Qingdao; RC, ambient seawater from Shidao Bay in Rongcheng.*

**TABLE 2:** Pairwise comparison of PERMANOVA analysis.

Group	Sample size	pseudo-*F*	*p*-value	*q*-value
Au vs. Cy	25	4.91	**0.001**	0.003
Au vs. Ne	27	5.75	**0.001**	0.003
Au vs. Rh	26	9.44	**0.001**	0.003
Au vs. QD	15	8.53	**0.003**	0.004
Cy vs. Ne	28	1.79	**0.003**	0.004
Cy vs. Rh	27	3.99	**0.001**	0.003
Cy vs. QD	16	4.96	**0.002**	0.004
Ne vs. Rh	29	2.05	**0.005**	0.005
Ne vs. RC	18	4.49	**0.001**	0.003
Rh vs. RC	17	5.43	**0.003**	0.004
QD vs. RC	6	5.23	0.115	0.115

***Note:** Abbreviation: Au, A. coerulea from Jiaozhou Bay in Qingdao; Cy, C. nozakii from Jiaozhou Bay in Qingdao; Ne, N. nomurai from Shidao Bay in Rongcheng; Rh, R. esculentum from Shidao Bay in Rongcheng; QD, ambient seawater from Jiaozhou Bay in Qingdao; RC, ambient seawater from Shidao Bay in Rongcheng. The values of p < 0.05 were bolded.*

### Core Bacterial Community Across Four Blooming Scyphozoans

To further analyze the bacterial variation, Alpha and Beta diversities of the bacterial communities of the three body parts of each scyphozoan were analyzed, represented by the Shannon index and PCoA analysis respectively. Results of Shannon indices demonstrated low divergence between the three body parts of each scyphozoan (Wilcoxon test, *p* > 0.05) (Supplementary Figure 2). PCoA analysis gave similar results (as shown in Supplementary Figure 3 and Supplementary Table 2). There was no significant difference between the three compartments of each scyphozoan taxa (PERMANOVA, *p* > 0.05).

**FIGURE 5:**
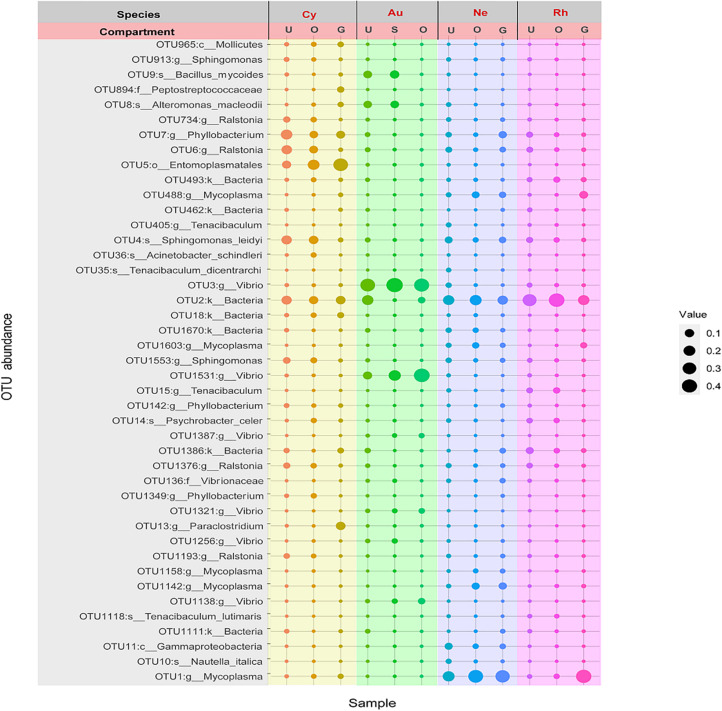
Bubble plot of core OTU members associated with four scyphozoan species with relative abundance greater than 1% in at least one body part of at least one scyphozoan species. **Note:** Abbreviations: Au, *A. coerulea* from Jiaozhou Bay in Qingdao; Cy, *C. nozakii* from Jiaozhou Bay in Qingdao; Ne, *N. nomurai* from Shidao Bay in Rongcheng; Rh, *R. esculentum* from Shidao Bay in Rongcheng; U, umbrella, O, oral arms, S, stomach and G, gonad. Bubble size indicates relative abundance.

In total, 57 OTUs were present in all scyphomedusa species and all compartments, which could represent core bacteria associated with the four scyphozoans (Figure 5). The core OTUs were composed mainly of *Mycoplasma*, *Vibrio*, *Ralstonia*, *Tenacibaculum*, *Shingomonas* and *Phyllobacterium* (Figure 5). These frequently occurring OTUs accounted for more than 80.99% of the core bacterial communities of each body part of each scyphomedusa species, except for the gonads (40.69%) and oral arms (65.67%) of *C. nozakii*, mainly because there were highly abundant unidentified *Entomoplasmatales* (OTU5) in these two compartments of *C. nozakii* (Figure 5).

### Functional Prediction of the 16S Genes Using FAPROTAX

**FIGURE 6:**
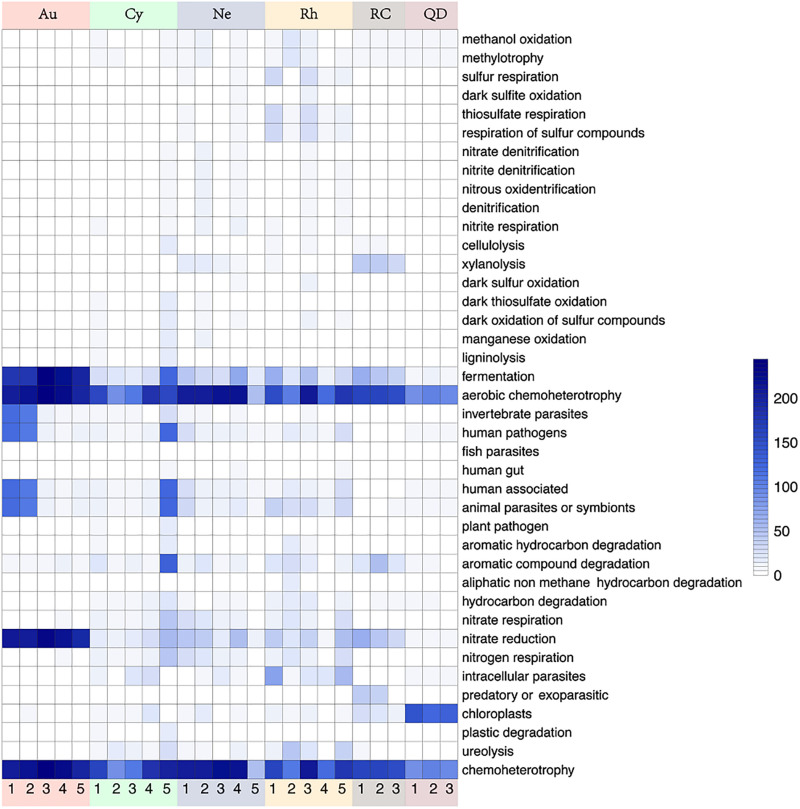
Heatmap of 40 most abundant functions based on FAPROTAX analysis in four scyphozoan-associated bacterial communities and free-living bacteria in surrounding seawater. **Note:** Abbreviations: Au, *A. coerulea* from Jiaozhou Bay in Qingdao; Cy, *C. nozakii* from Jiaozhou Bay in Qingdao; Ne, *N. nomurai* from Shidao Bay in Rongcheng; Rh, *R. esculentum* from Shidao Bay in Rongcheng; QD, ambient seawater from Jiaozhou Bay in Qingdao; RC, ambient seawater from Shidao Bay in Rongcheng. The values in the heatmap came from the square root transformation of the absolute abundance of OTUs contributing to functional groups after normalization.

Functional annotation of the genomic information was performed based on FAPROTAX. A total of 91 functional groups were obtained, comprising 8279 members (5011 unique members), and some OTUs were predicted to be included in multiple functional groups. 11 functional groups showed significant differences between scyphomedusa-associated bacterial communities and free-living bacterial communities in seawater in the top 40 FAPROTAX functional groups, of which 8 were significantly more abundant in scyphomedusae, and 3 (xylanolysis, predatory or exoparasitic, and chloroplasts) were significantly more abundant in seawater (Mann-Whitney *U*-test, *p* < 0.05) (Figure 6). The functional groups which were significantly more abundant in scyphomedusa-associated bacterial communities consisted of nitrogen cycle-related functions (nitrate respiration, and nitrogen respiration), and heterotrophic-dominated functions (aerobic chemoheterotrophy, chemoheterotrophy, invertebrate parasites, all human pathogens, human associated and animal parasites or symbionts) (Figure 6). Nitrate respiration mainly included *Vibrio*, *Bacillus*, *Arenibacter*, *Acinetobacter*, and *Stenotrophomonas*; the latter two genera were also the dominant players in nitrogen respiration (Supplementary Material 1). Aerobic chemoheterotrophy and chemoheterotrophy had the highest abundances of accumulated OTUs both in scyphomedusae and seawater, including *Vibrio*, *Bacillus*, *Stenotrophomonas*, *Arenibacter*, *Acinetobacter*, *Mycoplasma*, *Chryseobacterium*, *Tenacibaculum*, *Phyllobacterium*, *Sphingomonas*, etc. (Supplementary Material 1).

Twenty-one significantly distinct functional groups were tested from top 40 functional groups of the four scyphomedusa-associated bacterial communities. The significant differences between 15 of those 21 functional groups existed primarily between *A. coerulea* and *R. esculentum* (Kruskal-Wallis test, *p* < 0.05) (Figure 6 and Supplementary Table 3). The cumulative abundances of functional groups were usually lower in *A. coerulea* than the other three scyphomedusa species, with the exception of the following functional groups: fermentation, invertebrate parasites, aerobic chemoheterotrophy, chemoheterotrophy, and nitrate reduction, as well as plastic degradation. At least one of *Bacillus*, *Alteromonas*, family Vibrionaceae, and *Vibrio* held a larger proportion in the assigned OTUs of the former five functional groups, and these OTUs happened to be more abundant in *A. coerulea* than in the other three scyphomedusa species (Figures 2, 6, and Supplementary Table 3, Supplementary Material 1). Plastic degradation was richer in *C. nozakii*, inferred to be related to the high relative abundance of *Pseudomonas* in the *C. nozakii*-associated bacterial communities (Figures 2, 6, and Supplementary Table 3, Supplementary Material 1). In addition, cellulolysis, aromatic hydrocarbon degradation, hydrocarbon degradation and ureolysis functional groups were well-represented among *C. nozakii*-associated bacteria. Interestingly, these functional groups were all related to material decomposition (Figure 6 and Supplementary Table 3). The two functional groups xylanolysis and nitrate respiration were most abundant in *N. nomurai* among the four scyphomedusa taxa (Figure 6 and Supplementary Table 3). *R. esculentum* had the highest cumulative abundances of 13 functional groups, covering the sulfur cycle, compound degradation and parasites, indicating that the FAPROTAX functions of *R. esculentum*-associated bacteria communities were more diverse and enriched than those of the other scyphomedusae.

### Potentially Pathogenic Bacteria Associated With Four Blooming Scyphozoans

**FIGURE 7:**
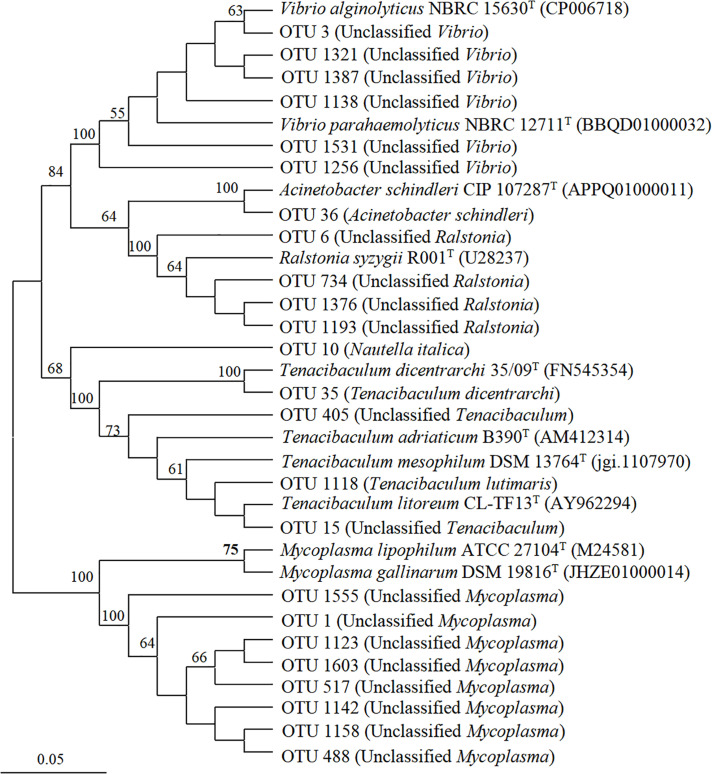
Neighbor-joining phylogenetic tree generated from the V4-V5 region of the 16S rRNA gene for scyphozoan-associated bacterial communities and reference sequences of identified pathogenic bacteria. The bootstrap was performed with 1000 replicates. Operational taxonomic units are from this study. Reference sequences include NCBI accession numbers.

Six genera of potentially pathogenic bacteria were detected in the scyphozoan-associated bacterial communities based on phylogenetic analysis: *Vibrio*, *Mycoplasma*, *Ralstonia*, *Tenacibaculum*, *Nautella*, and *Acinetobacter* (Figure 7). Potentially pathogenic *Vibrio*, clustered with *Vibrio alginolyticus* and *Vibrio parahaemolyticus*, was the dominant genus in *A. coerulea* (> 48.43%), and its relative abundance was highest in the oral arms of *A. coerulea* (91.62%), followed by the umbrellas of *N. nomurai* (2.71%) and *R. esculentum* (2.06%). *Mycoplasma* was the second most abundant potentially pathogenic genus in the scyphozoan-associated bacterial communities, maintaining high relative abundances in all three body components (umbrella, oral arms, and gonad) of the other three scyphomedusae (*C. nozakii*, *N. nomurai*, and *R. esculentum*), and was the most abundant bacteria in the gonads of *R. esculentum* (65.93%), as well as the oral arms (57.14%), gonads (49.92%), and umbrellas (26.11%) of *N. nomurai*. *Ralstonia*, closely related to *Ralstonia syzygii*, was the most widely distributed potentially pathogenic genus, with relatively high abundances in all four scyphomedusa taxa, among which the relative abundances were particularly high in the umbrellas (23.78%) and oral arms (13.77%) of *C. nozakii*.

*N. nomurai* harbored the most diverse potential pathogens, with 5 genera (*Vibrio*, *Mycoplasma*, *Ralstonia*, *Nautella* and *Tenacibaculum*). *R. esculentum* was associated with 4 potentially pathogenic genera (*Vibrio*, *Mycoplasma*, *Ralstonia*, and *Tenacibaculum*). *C. nozakii* contained 3 genera of potentially pathogenic bacteria (*Mycoplasma*, *Ralstonia* and *Acinetobacter*), of which *Acinetobacter* was only present in the oral arms in high relative abundance (1.79%), while *A. coerulea* was associated with *Vibrio* and *Ralstonia*. Genera *Tenacibaculum*, *Nautella*, and *Acinetobacter* were clearly identified at the species level: fish pathogens *Tenacibaculum dicentrarchi* and *Tenacibaculum lutimaris*, red alga pathogen *Nautella italica*, and human pathogen *Acinetobacter schindleri* (Figure 7).

## Discussion

Jellyfish are conspicuous marine zooplankton that play critical roles in the exchange of material and energy in marine ecosystems (Riemann et al., 2006; Pitt et al., 2009; West et al., 2009; Condon et al., 2011; Blanchet et al., 2015; Chelsky et al., 2015; Sweetman et al., 2016; Guy-Haim et al., 2020). The four scyphomedusae in this study (*A. coerulea*, *C. nozakii*, *N. nomurai*, and *R. esculentum*) can all reach considerable biomass in coastal waters of China during June to September (Dong et al., 2010, 2014; Zhang et al., 2012; Wang P.P. et al., 2020). Blooming jellyfish may convert large amounts of carbon into gelatinous biomass, which may limit the transfer of carbon in the food web (Condon and Steinberg, 2008; Sweetman et al., 2016). Organic and inorganic matters released by jellyfish could influence microbial nutrient cycling and alter microbial structure and function (Riemann et al., 2006; Condon et al., 2011; Guy-Haim et al., 2020). Therefore, bacterial communities associated with the blooming cnidarian jellyfish were speculated to be major participants in the biogeochemical cycling of carbon, nitrogen, sulfur, and phosphorus (Lee et al., 2018) and represent key vectors of potentially bacterial pathogens (Ferguson et al., 2010; Schuett and Doepke, 2010; Delannoy et al., 2011; Basso et al., 2019; Clinton et al., 2020).

### The Composition and Potential Functions of the Bacterial Communities Associated With the Four Scyphozoans

Scyphomedusa-associated bacterial communities presented different composition and lower diversities compared with the free-living bacterial communities in the ambient seawater, consistent with previous studies (Weiland-Bräuer et al., 2015; Daley et al., 2016; Kramar et al., 2019; Daniel and Ana, 2020). Most previous studies on scyphozoan-associated microbiomes focused on one single scyphozoan species (e.g., Cortés-Lara et al., 2015; Weiland-Bräuer et al., 2015; Viver et al., 2017; Lee et al., 2018; Basso et al., 2019; Kramar et al., 2019; Stabili et al., 2020), with few studies investigating the differences between the bacteria associated with various scyphozoan species. In the present study, the composition and structure of the microbiomes associated with four scyphomedusae were compared and found to be significantly different from each other, indicating that the scyphomedusa-associated bacteria were scyphozoan taxa-specific (as Cleary et al., 2016; Hao et al., 2019; and Daniel and Ana, 2020 reported). The *A. coerulea*-associated bacterial communities were dominated by *Vibrio*, as has previously been shown for the communities associated with *A. aurita* (Weiland-Bräuer et al., 2015; Kramar et al., 2019; Jaspers et al., 2020). The bacterial communities associated with *N. nomurai* and *R. esculentum* (family Rhizostomatidae) were similarly dominated by a single genus, *Mycoplasma*, which was consistent with previous results for *R. pulmo* (family Rhizostomatidae) (Basso et al., 2019; Stabili et al., 2020). Interestingly, three bacterial genera presented relatively similar abundances associated with *C. nozakii* including *Shingomonas*, *Phyllobacterium*, and *Ralstonia*. Schuett and Doepke (2010) first reported both *Cyanea* species (*C. capillata* and *C. lamarckii*) which were dominated by Gammaproteobacteria, exhibiting > 97% similarity to the *Vibrio* group based on culturing and culture-independent approaches. This might be due to the different methodologies applied, e.g., preculturing step, increasing the bottle effects and affecting the relevance of the resulting data.

A total of 57 core OTUs were identified in our study of the bacterial communities of the scyphozoan body parts, predominantly genera *Mycoplasma*, *Vibrio*, *Ralstonia*, *Tenacibaculum*, and *Phyllobacterium*. Comparison of the core bacteria in this study with previous reports of scyphozoan-associated bacteria found several commonalities. Several studies found *Mycoplasma* to be the prevailing taxa in the microbiomes of the scyphozoans *A. aurita* (Weiland-Bräuer et al., 2015; Daley et al., 2016; Jaspers et al., 2020), and *R. pulmo* (Basso et al., 2019; Stabili et al., 2020), similar to the present study. *Vibrio* has previously been found to be the dominant genus in *A. aurita* (Weiland-Bräuer et al., 2015; Kramar et al., 2019; Jaspers et al., 2020), *A. coerulea* (Chen et al., 2020), *R. pulmo* (Basso et al., 2019; Stabili et al., 2020), *Cotylorhiza tuberculata* (Cortés-Lara et al., 2015), *C. lamarckii* (Schuett and Doepke, 2010), and *C. capillata* (Schuett and Doepke, 2010; Clinton et al., 2020), and was abundant in *A. coerulea*, *N. nomurai* and *R. esculentum* in this study. Genus *Tenacibaculum* was dominant in *N. nomurai* and *R. esculentum* in this study, and has been suggested as a key part of the bacterial communities of *A. aurita* (Jaspers et al., 2020), *C. tuberculata* (Cortés-Lara et al., 2015; Viver et al., 2017) and *P. noctiluca* (Delannoy et al., 2011). These common genera might occupy important micro-niches in the bacterial communities of scyphozoans.

The FAPROTAX method is most suitable for annotating and predicting ecological functions such as biogeochemical cycling processes (Louca et al., 2016). Our results suggested that some bacteria associated with the scyphozoans, such as *Vibrio*, *Bacillus*, *Arenibacter*, *Acinetobacter* and *Stenotrophomonas*, might play important roles in marine biogeochemical cycles. For example, *Vibrio* was involved in the nitrogen cycle, while *Bacillus* (mainly *B. megaterium*) was involved in the nitrogen and sulfur cycle. Such bacteria are likely to provide their hosts with unavailable nutrients (Lee et al., 2018). Ammonia-, nitrite- and sulfur-oxidizing microbes may be scavengers of toxic ammonia, nitrite and sulfide in the host (Tian et al., 2016). Moreover, during the decomposition of jellyfish after blooms, certain bacteria (e.g., Vibrionaceae) could grow rapidly, causing the accumulation of a large amount of inorganic nutrients and participating in the regeneration of elements (Tinta et al., 2012).

The genera *Alteromonas*, *Tenacibaculum*, *Mycoplasma* and *Sphingomonas* had only a single function, chemoheterotrophy, in the present study. Of the functional groups identified, chemoheterotrophy, including aerobic chemoheterotrophy and chemoheterotrophy, had the highest cumulative abundance of OTUs, both in scyphozoans and seawater, indicating that they played key roles both in scyphozoan-associated and free-living bacterial communities. Chemoheterotrophy has been reported as an essential ecological function of bacterioplankton and sediments (Zeng et al., 2014; He et al., 2020). Some scyphomedusa-associated bacteria displayed pollutant degradation including *Alteromonas* (Bakunina et al., 2013; Lopardo and Urakawa, 2019), *Sphingomonas* (Walayat et al., 2018; Carmen Garcia et al., 2019; Menon et al., 2019; Yun et al., 2019) and *Ralstonia* (Wang J. et al., 2020). It has been reported that scyphozoans are highly tolerant of certain pollutants (e.g., heavy metals and crude oil), and some of the most toxic PAHs of crude oil can be bioaccumulated in gelatinous zooplankton and potentially be transferred through the food web to contaminate apex predators (Almeda et al., 2013; Lucas and Horton, 2014). We speculated that these bacteria might be related to the pollution-tolerance of scyphomedusae. This might indicate that the jellyfish-associated bacterial communities plays an important ecological function in assisting longer tolerant of jellyfish in the environment pollution.

### Potentially Pathogenic Bacteria Associated With the Four Scyphozoan Microbiomes

Since we applied the function prediction based on FAPROTAX which mainly focused on the biogeochemical cycle, the potential pathogens we mentioned here mainly focused on human pathogens. The database for pathogens of marine economic species is still missing. Here, we specifically discussed the potentially pathogenic bacteria associated with four scyphomedusa taxa in this study based on phylogenetic analysis. It has been shown that certain jellyfish-associated microbial communities contain pathogens, such as *Tenacibaculum*, *Vibrio*, *Pseudomonas*, *Aeromonas*, *Photobacterium*, *Flavobacterium*, *Mycoplasma*, and *Chryseobacterium*, among others (Ferguson et al., 2010; Schuett and Doepke, 2010; Delannoy et al., 2011; Cortés-Lara et al., 2015; Småge et al., 2017; Basso et al., 2019; Clinton et al., 2020; Jaspers et al., 2020; Stabili et al., 2020). Some researchers have expressed concern that jellyfish may threaten aquaculture and human health by spreading pathogens (Ferguson et al., 2010; Schuett and Doepke, 2010; Delannoy et al., 2011).

In this study, high relative abundances of *Vibrio* associated with *A. coerulea, N. nomurai* and *R. esculentum* were closely related to *V. alginolyticus* and *V. parahaemolyticus*. *V. alginolyticus* and *V. parahaemolyticus* have been determined to infect marine animals (e.g., fish, shrimp, shellfish) and even humans, and are common pathogens in aquaculture (Joshi et al., 2014; Lee et al., 2015; Escobedo-Hinojosa and Pardo-Lopez, 2017; Aly et al., 2021; Hsu et al., 2021). Genus *Mycoplasma* has been reported to be associated with algae (Altamiranda et al., 2011; Davis et al., 2013), as well as invertebrates such as bivalves (Fernandez-Piquer et al., 2012), crustaceans (Liang et al., 2011), ctenophores (Hao et al., 2015), and other cnidarians (Cortés-Lara et al., 2015; Weiland-Bräuer et al., 2015; Viver et al., 2017). To date, the function of *Mycoplasma* bacteria on gelatinous zooplankton remains unknown. Some *Mycoplasma* species are classified as common pathogens, such as *M. mobile*, *M. penetrans*, *M. pneumoniae* (Escobedo-Hinojosa and Pardo-Lopez, 2017). In this study, abundant *Mycoplasma* were detected in *N. nomurai*, *R. esculentum* and *C. nozakii*. Although we did not find homologous certified pathogens of *Mycoplasma* in this study, we cannot rule out the possibility that the unclassified *Mycoplasma* in this study included pathogenic species.

Genus *Tenacibaculum* currently includes a total of 21 species pathogenic to fish (Habib et al., 2014). *T. dicentrarchi* and *T. lutimaris* in this study was mainly associated with *N. nomurai* and *R. esculentum*, and is known to be pathogenic to fish (Avendano-Herrera et al., 2016; Slinger et al., 2020). Unclassified *Tenacibaculum* was clustered with two other fish pathogens, *T. adriaticum* and *T. litoreum*, which may pose a threat to fish populations and aquaculture industry (Habib et al., 2014; Fernandez-Alvarez et al., 2017). Moreover, *Tenacibaculum* spp. may be a key part of the digestive system of jellyfish, playing an important role in their immune defense and nutrition (Ferguson et al., 2010; Cortés-Lara et al., 2015).

*Ralstonia* was the most widely distributed potentially pathogenic genus among the four scyphomedusa species. *Ralstonia* spp. are non-fermenting gram-negative bacteria that have recently emerged as opportunistic pathogens (Alasehir et al., 2020), with *Ralstonia picketti*, *R. mannitolilytica* and *R. insidiosa* implicated particularly in nosocomial infections in immunocompromised patients (Fang et al., 2019). *R. syzygii*, one of the phylotypes of *R. solanacearum*, is believed to cause plant disease (Etminani et al., 2020). The widespread distribution of *Ralstonia* in various scyphomedusa taxa may present a risk for the spread of human and foodborne pathogens.

Genus *Nautella*, identified in the umbrella of *N. nomurai*, was dominated by *N. italica*, an etiological agent of bleaching in the red alga *Delisea pulchra* (Case et al., 2011; Gardiner et al., 2017; Hudson et al., 2018). The most abundant species of genus *Acinetobacter*, detected in the oral arms of *C. nozakii*, was *A. schindleri*, a human pathogen (McGann et al., 2013; Jani et al., 2019).

Due to the short amplified sequence and lack of verification by culture methods in our study, the pathogenic bacteria discussed in this study are only potential pathogens. To determine its pathogenicity and virulence, it is necessary to carry out long-range amplification sequencing, and isolation and culture of target bacteria. Some researchers put forward the hypothesis that scyphomedusae, carrying potential pathogens, might drift with marine currents over long distances and spread to large sea areas (Dong et al., 2010; Tinta et al., 2019). Outbreaks of the four scyphomedusae in this study commonly occur in areas of Chinese coastal waters with large aquaculture industries. China, as the largest exporter of aquatic products in the world, uses vast areas of sea for aquaculture, diverse aquaculture models (e.g., raft culture, cage culture, and pond culture) and numerous species of aquaculture organisms (e.g., fish, shellfish, shrimp, and sea cucumber) (Cao et al., 2015; Zhou et al., 2020). In such conditions, high occurrence of potentially pathogenic bacteria in common scyphomedusae is extremely concerning. Living scyphomedusae may pass pathogenic bacteria to other organisms including humans through contact or attack, causing bacterial diseases and even death (Basso et al., 2019; Clinton et al., 2020). During scyphomedusa degradation, certain pathogenic bacteria may be released into the surrounding seawater, increasing the risk of infection of other organisms in the surrounding area (Tinta et al., 2012). Furthermore, jellyfish carcasses might trigger or support growth of potential pathogens in ambient seawater (Dinasquet et al., 2012). Farmed organisms may suffer greater dangers compared to others due to their high population densities and habitats that overlap with blooming scyphomedusae. This has the potential to cause immense socio-economic losses to the aquaculture industry.

## Data Availability Statement

The raw sequence data generated herein have been uploaded to NCBI’s Sequence Read Archive (SRA) under BioProject accession number PRJNA718816.

## Author Contributions

SP did the formal analysis, data analysis, writing – original draft, and writing – review and editing. WH did the formal analysis, data analysis, and writing – review and editing. YL did the formal analysis and data analysis. LW and TS did the investigation and sample collection. JZ did the conceptualization and funding acquisition. ZD did the conceptualization, sample collection, data analysis, funding acquisition, and writing – review and editing. All authors contributed to the article and approved the submitted version.

## Conflict of Interest

The authors declare that the research was conducted in the absence of any commercial or financial relationships that could be construed as a potential conflict of interest.

## Footnotes

1 http://www.arb-silva.de/
