# Supplementary material for: Bacterial Communities Associated With Four Blooming Scyphozoan Jellyfish: Potential Species-Specific Consequences for Marine Organisms and Humans Health
Source: Front Microbiol. 2021 May 5;12:647089. doi: 10.3389/fmicb.2021.647089 (PMC8131558; doi:10.3389/fmicb.2021.647089)
Supplement: Supplementary file 1 [file Table_1.doc]

**List of supplementary Figures:**

**Figure S1**: Shannon curve of scyphozoan-associated bacteria and free-living bacteria in surrounding seawater.

**Figure S2:** Boxplot of Shannon indices of bacteria associated with various body parts of four scyphomedusae. **Note:** Abbreviation: Au - *A. coerulea* from Jiaozhou Bay in Qingdao; Cy - *C. nozakii* from Jiaozhou Bay in Qingdao; Ne - *N. nomurai* from Shidao Bay in Rongcheng; Rh - *R. esculentum* from Shidao Bay in Rongcheng; U - umbrella, O - oral arms, S - stomach and G - gonad. Significant differences were tested by Wilcoxon test, *: *p* < 0.05, ns: no significant difference.

**Figure S3:** Unweighted unifrac PCoA plot of bacteria associated with various compartments of four scyphomedusae. **Note:** Abbreviation: Au - *A. coerulea* from Jiaozhou Bay in Qingdao; Cy - *C. nozakii* from Jiaozhou Bay in Qingdao; Ne - *N. nomurai* from Shidao Bay in Rongcheng; Rh - *R. esculentum* from Shidao Bay in Rongcheng; U - umbrella, O - oral arms, S - stomach and G - gonad.

**List of supplementary Tables:**

**Table S1**: Alpha diversity indices of bacterial communities. **Note:** The means of six indices are shown. Abbreviation: Au - *A. coerulea* from Jiaozhou Bay in Qingdao; Cy - *C. nozakii* from Jiaozhou Bay in Qingdao; Ne - *N. nomurai* from Shidao Bay in Rongcheng; Rh - *R. esculentum* from Shidao Bay in Rongcheng; QD - ambient seawater from Jiaozhou Bay in Qingdao; RC - ambient seawater from Shidao Bay in Rongcheng; U - umbrella, O - oral arms, S - stomach and G - gonad.

**Table S2**: Pairwise comparison of PERMANOVA analysis of bacteria associated with various body parts of four scyphomedusae. **Note**: Abbreviation: Au - *A. coerulea* from Jiaozhou Bay in Qingdao; Cy - *C. nozakii* from Jiaozhou Bay in Qingdao; Ne - *N. nomurai* from Shidao Bay in Rongcheng; Rh - *R. esculentum* from Shidao Bay in Rongcheng; U - umbrella, O - oral arms, S - stomach and G - gonad.

**Table S3**: Twenty-one functional groups with significant differences among the four scyphozoan bacterial communities within the most abundant 40 FAPROTAX functional groups. **Note:** Abbreviation: Au - *A. coerulea* from Jiaozhou Bay in Qingdao; Cy - *C. nozakii* from Jiaozhou Bay in Qingdao; Ne - *N. nomurai* from Shidao Bay in Rongcheng; Rh - *R. esculentum* from Shidao Bay in Rongcheng. The statistical test method was Kruskal-Wallis test, and *p* values in the second column were the results of multiple comparisons. The *p* values in the third column were the results of pairwise comparisons, showing only the pairs with significant differences.

**
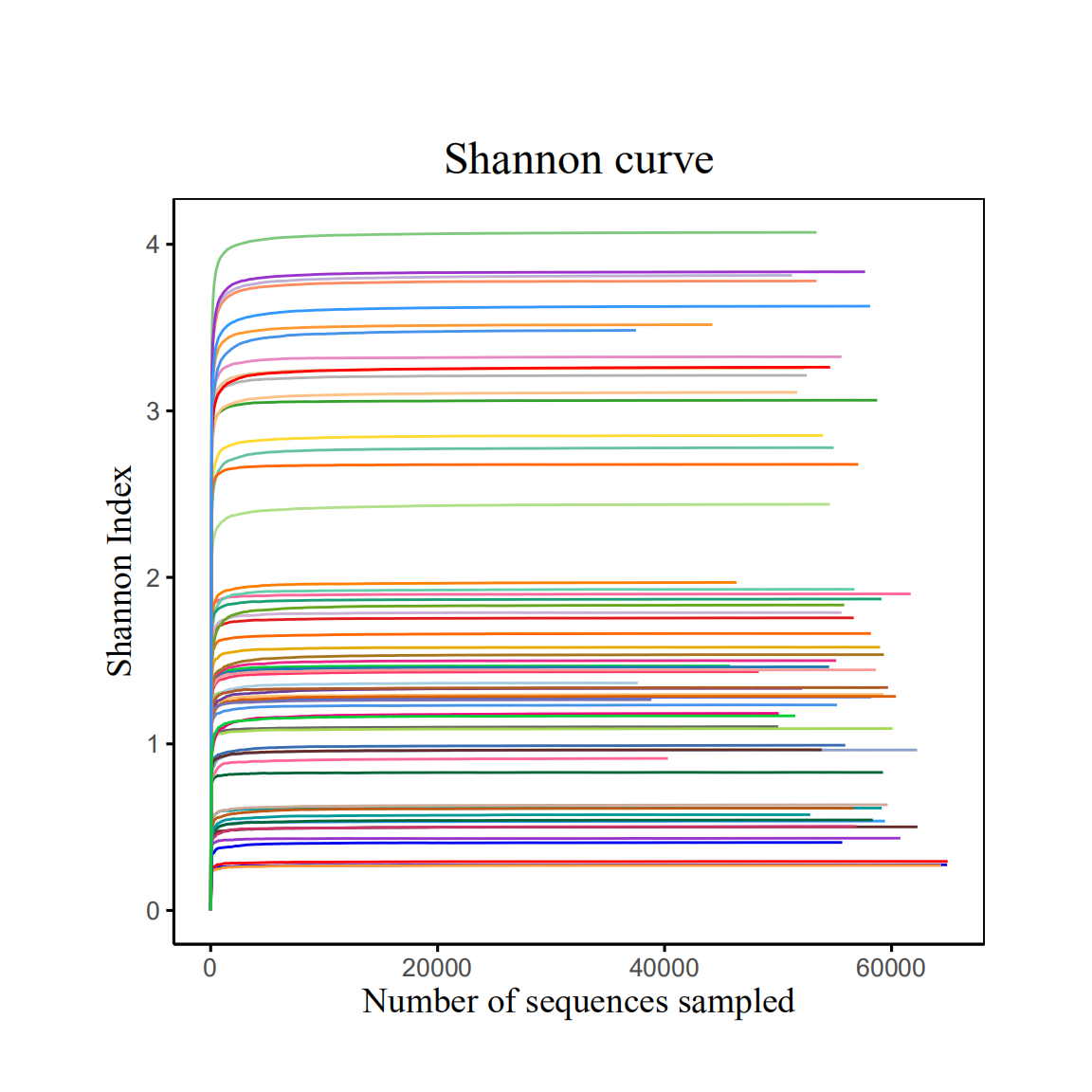
**

**Figure S1**


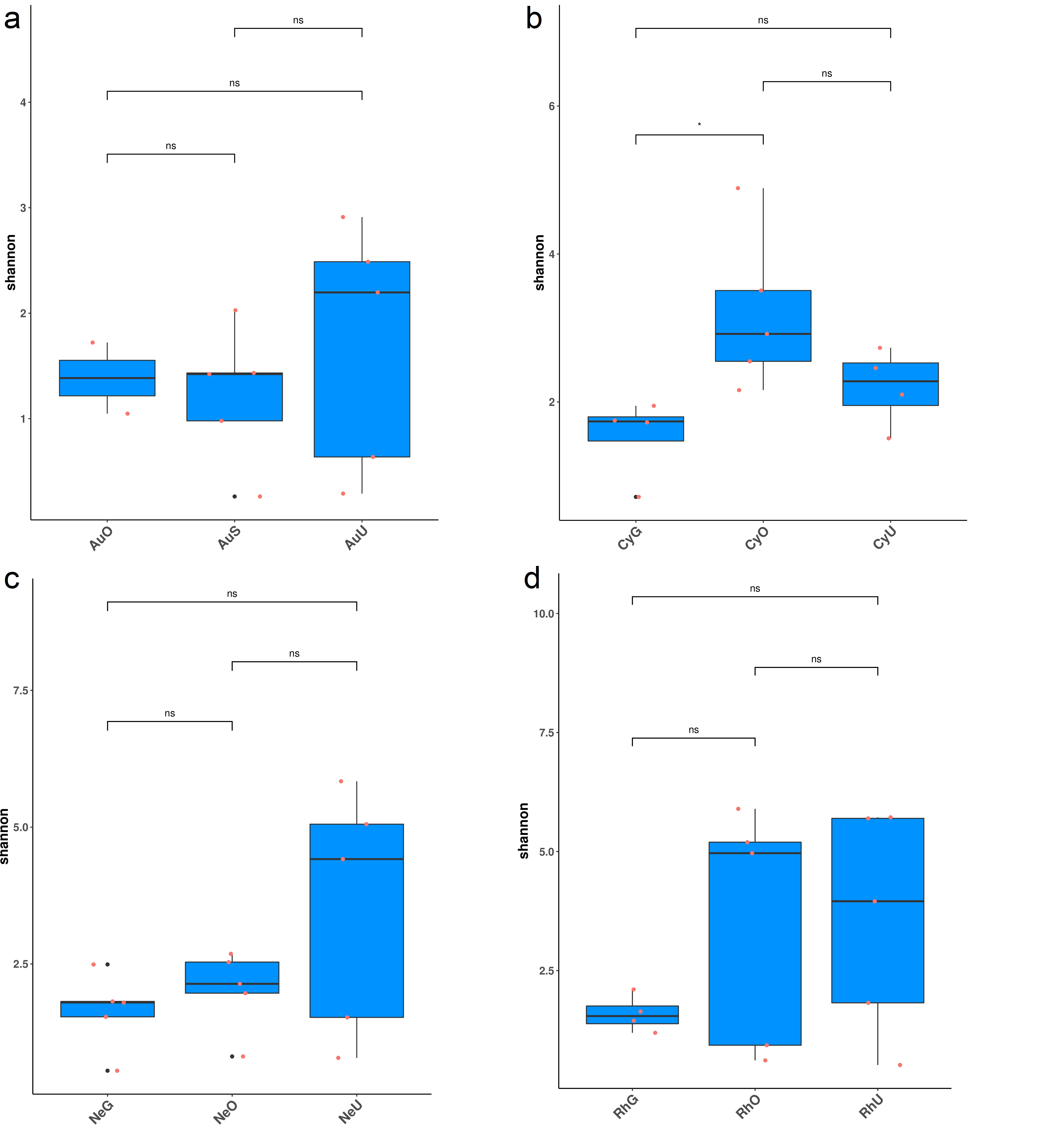


**Figure S2**


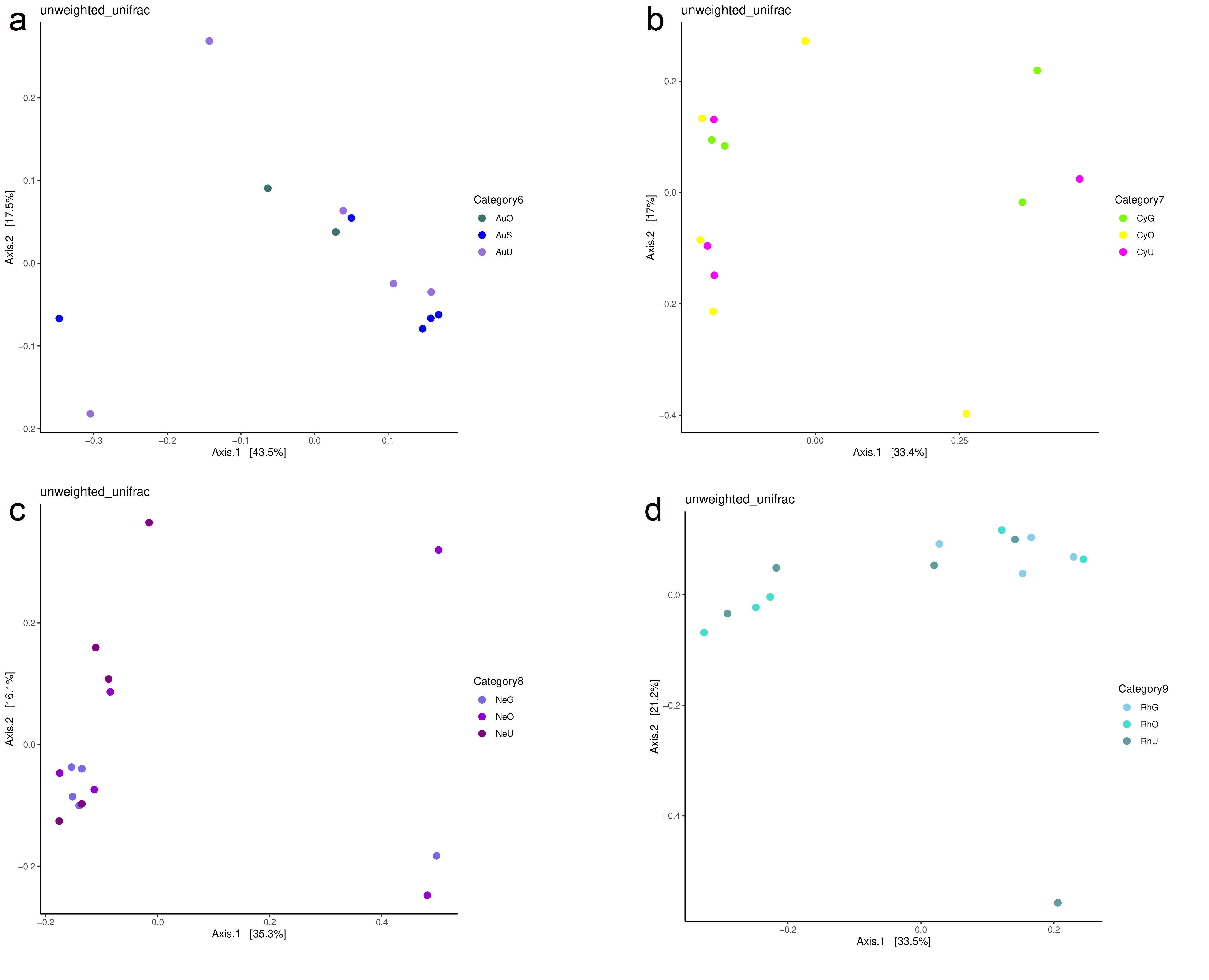


**Figure S3**

**Table S1. Alpha diversity indices of bacterial communities**

| Sample | Observed OTUs | Chao1 | ACE | Shannon | Simpson | Goods coverage |
| --- | --- | --- | --- | --- | --- | --- |
| AuU | 127.000±32.220 | 170.037±24.413 | 178.382±23.535 | 2.107±0.986 | 0.541±0.252 | 0.999 |
| AuO | 105.000±3.500 | 149.463±1.287 | 154.097±7.000 | 1.917±0.188 | 0.605±0.035 | 0.999 |
| AuS | 105.000±33.347 | 138.328±30.015 | 156.687±33.849 | 1.450±0.722 | 0.440±0.247 | 0.999 |
| CyU | 185.000±42.889 | 218.322±36.781 | 219.392±41.091 | 3.137±0.278 | 0.753±0.080 | 0.999 |
| CyG | 171.000±84.925 | 221.710±84.568 | 236.888±91.639 | 2.104±0.990 | 0.537±0.144 | 0.999 |
| CyO | 236.000±28.376 | 269.718±48.697 | 274.980±50.602 | 3.649±0.825 | 0.792±0.223 | 0.999 |
| QD | 327.000±15.585 | 364.318±19.828 | 383.719±17.022 | 4.940±0.204 | 0.932±0.012 | 0.998 |
| NeU | 266.000±94.239 | 288.965±87.141 | 295.757±82.449 | 3.976±1.832 | 0.730±0.260 | 0.999 |
| NeG | 195.000±49.978 | 235.054±61.086 | 239.129±63.628 | 2.902±0.546 | 0.698±0.107 | 0.999 |
| NeO | 227.000±69.382 | 265.974±72.484 | 257.232±68.581 | 3.019±0.814 | 0.678±0.156 | 0.999 |
| RhU | 288.000±107.103 | 326.993±112.745 | 323.672±103.188 | 4.070±1.546 | 0.785±0.200 | 0.999 |
| RhG | 211.000±79.966 | 262.686±90.012 | 275.037±91.344 | 2.477±0.246 | 0.638±0.103 | 0.999 |
| RhO | 335.000±145.105 | 357.526±142.763 | 367.183±133.969 | 3.847±2.174 | 0.679±0.317 | 0.999 |
| RC | 405.000±33.357 | 436.496±34.275 | 454.580±29.479 | 5.542±0.534 | 0.937±0.025 | 0.998 |

**Table S2 Pairwise comparison of PERMANOVA analysis of bacteria associated with various body parts of four scyphomedusae**

| Group | Sample size | pseudo-F | *p*-value | *q*-value |
| --- | --- | --- | --- | --- |
| *Aurelia coerulea* | | | | |
| AuO vs. AuS | 7 | 0.879 | 0.563 | 0.808 |
| AuO vs. AuU | 7 | 0.788 | 0.808 | 0.808 |
| AuS vs. AuU | 10 | 0.990 | 0.410 | 0.808 |
| *Cyanea nozakii* | | | | |
| CyG vs. CyO | 9 | 1.258 | 0.169 | 0.267 |
| CyG vs. CyU | 8 | 1.289 | 0.178 | 0.267 |
| CyO vs. CyU | 9 | 0.650 | 0.992 | 0.992 |
| *Nemopilema nomurai* | | | | |
| NeG vs. NeO | 10 | 0.780 | 0.751 | 0.751 |
| NeG vs. NeU | 10 | 0.993 | 0.482 | 0.723 |
| NeO vs. NeU | 10 | 1.094 | 0.420 | 0.723 |
| *Rhopilema esculentum* | | | | |
| RhG vs. RhO | 9 | 1.343 | 0.168 | 0.2835 |
| RhG vs. RhU | 9 | 1.162 | 0.189 | 0.2835 |
| RhO vs. RhU | 10 | 0.674 | 0.952 | 0.952 |

**Table S3 Twenty-one functional groups with significant differences among the four scyphozoan bacterial communities within the most abundant 40 FAPROTAX functional groups.**

| Functional groups | *p* value | Pairwise comparison (*p* value) |
| --- | --- | --- |
| Sulfur respiration | 0.001 | Au < Rh (0.001); Cy < Rh (0.027) |
| Dark sulfite oxidation | 0.036 | Au < Rh (0.034) |
| Thiosulfate respiration | 0.001 | Au < Rh (0.001); Cy < Rh (0.021) |
| Respiration of sulfur compounds | 0.001 | Au < Rh (0.001); Cy < Rh (0.021) |
| Cellulolysis | 0.01 | Au < Cy (0.026); Au < Rh (0.022) |
| Xylanolysis | 0.001 | Au < Ne (0.003); Cy < Ne (0.036); Au < Rh (0.043) |
| Dark sulfur oxidation | 0.036 | Au < Rh (0.034) |
| Fermentation | 0.013 | Au > Cy (0.027); Au > Rh (0.049) |
| Aerobic chemoheterotrophy | 0.034 | Au > Cy (0.049) |
| Invertebrate parasites | 0.017 | Au > Rh (0.011) |
| Aromatic hydrocarbon degradation | 0.007 | Au < Cy (0.043); Au < Rh (0.030) |
| Aliphatic non methane hydrocarbon degradation | 0.014 | Au < Rh (0.008) |
| Hydrocarbon degradation | 0.005 | Au< Cy (0.021); Au < Rh (0.009) |
| Nitrate respiration | 0.024 | Au < Ne (0.045) |
| Nitrate reduction | 0.012 | Au > Cy (0.018) |
| Nitrogen respiration | 0.026 | Pairs with no significant difference |
| Intracellular parasites | 0.007 | Au < Rh (0.004) |
| Predatory or exoparasitic | 0.011 | Au < Rh (0.009) |
| Plastic degradation | 0.037 | Cy > Ne (0.033) |
| Ureolysis | 0.009 | Au < Cy (0.047); Au < Rh (0.010) |
| Chemoheterotrophy | 0.045 | Pairs with no significant difference |
